# Assessing the energy load and environmental footprint of potash fertilizer production in Iran

**DOI:** 10.1371/journal.pone.0313129

**Published:** 2024-11-07

**Authors:** Saeid Shahvarooghi Farahani, Hossein Zamanifard, Morteza Taki

**Affiliations:** Department of Agricultural Machinery and Mechanization Engineering, Agricultural Sciences and Natural Resources University of Khuzestan, Mollasani, Iran; ICAR - IIFSR: ICAR - Indian Institute of Farming Systems Research, INDIA

## Abstract

The goal of this research was to analyze the energy and environmental impact of KCL and K_2_SO_4_ production and provide recommendations for enhancing energy efficiency and environmental practices. Data was collected through face-to-face interviews at two potash plants and the CML methodology was employed to assess impact categories. Inventory data for production inputs were sourced from the Ecoinvent, BUWAL 250, and LCA Food DK databases within the Simapro 8.03.14 software. The results showed that the production of one ton of K_2_O as KCL and K_2_SO_4_, required 7080.82 and 15691.5 MJ, respectively. Electricity accounted for 52.96% of energy input in KCL production, whereas fuel oil constituted 38.39% in K_2_SO_4_ production. Energy ratios, energy productivity and specific energy for K_2_SO_4_ was 0.40, 0.06 kgMJ^-1^, and 15.6 MJkg^-1^, while corresponding indices for KCL were 0.90, 0.14 kgMJ^-1^ and 7.08 MJkg^-1^, respectively. In KCL production, electricity had eight impact categories, while the use of KCL as a raw material in K_2_SO_4_ production had significant effects on seven impact categories. Considering the vast and unoccupied space available in Iran’s great desert, where the KCL plant is situated, the installation of a photovoltaic power station near the plant could greatly enhance energy efficiency and reduce emissions.

## 1 Introduction

The agricultural sector is responsible for approximately 10–12% of Greenhouse Gas (GHG) emissions worldwide [1]. Given that the global population is expected to reach 10 billion by 2050, the demand for agricultural products and the resulting environmental impacts of the agricultural sector are likely to increase [2]. So, it is essential to apply scientific and technical methods and procedures to control and reduce the environmental impacts of the agricultural sector [3]. Adopting management techniques to use fertilizers and machinery more efficiently, exploring alternatives to reduce the use of pesticides, and using renewable energies instead of fossil fuels are necessary steps to reduce environmental impacts [4]. Additionally, since indirect emissions (off-farm) in agriculture are responsible for the majority of impacts on the environment, producing more eco-friendly agricultural inputs, such as fertilizers and pesticides, is an efficient way to reduce the environmental impacts of the agricultural sector. In open-field agriculture, 55% of total energy inputs come from indirect sources. The production of chemical fertilizers results in significant environmental impacts due to the high amount of energy consumption involved. Chemical fertilizers account for about 38% of GHG emissions in the agricultural sector [5].

Some studies have reported that chemical fertilizers production accounts for approximately 57% of agricultural inputs in China [6, 7].

The production of fertilizers heavily relies on fossil fuels as fuel and feedstock, which resulted in the generation of at least 3 tons of CO_2_ per ton of fertilizer [8, 9]. However, it is expected that improved technologies, including lower energy-consuming separation and catalytic technologies, will significantly reduce the environmental impacts of fertilizer production in the future [8]. Potassium is a crucial nutrient for plant growth, along with nitrogen and phosphorus. It is essential for agriculture as it enhances water retention, nutrient value, disease resistance, taste, and yield. More than 90% of potash production is currently used as fertilizer [10, 11].

Efficient use of energy in agriculture is a critical step towards achieving sustainability, as it reduces input costs, fossil fuel consumption, and GHG emissions [12]. Energy analysis can help to calculate the total energy required to produce a specific amount of a product or service and identify ways to reduce the total energy requirement. Several studies have examined energy use in fertilizers production, including NPK-15:8:15 [13], organic fertilizer production [14], nitrogen fertilizer [15], ammonia [16], and single superphosphate [17].

Life Cycle Assessment (LCA) is a useful methodological tool for evaluating the environmental impacts associated with the life cycle of a product or service [18]. The use of LCA methodology can help decision-makers improve sustainability in industries [19]. Several studies in the literature have employed LCA to investigate environmental emissions in fertilizers production, including complex fertilizers, synthetic fertilizers [8], ammonium nitrate, ammonium sulfate, single superphosphate, triple superphosphate, and diammonium phosphate and nitrogen and compound fertilizers [20], as well as phosphate fertilizer and diammonium phosphate [21].

**Table 1** provides an overview of some studies that used energy analysis and LCA to evaluate various agricultural products. However, there are relatively few LCA studies that have assessed the environmental burdens of potash fertilizer, with studies by Nemecek et al. (2007) [22] and Chen et al. (2018) [23] being notable examples. Also, these few studies barely covered all the production process details and their results indicate that environmental burdens associated with potash production depend on potash mining methods. So, it is crucial to conduct further LCA studies on potash fertilizer as it is a significant source of potassium in agriculture and the environmental burdens associated with the production process could be completely different due to the way in which potash is extracted.

**Table 1:** The summary of some studies that used energy analysis and LCA to evaluate agricultural productions and industries related to agriculture.

References	Product	Energy output	LCA	Energy indexes			
				Energy ratio	Energy productivity	Specific energy	Net energy gain
[24]	Strawberry	75,933	✓	0.15	0.08	12.98	-442,914
[25]	Barley	43,345.00	✓	3.22	151.64	×	29.9
[26]	Biofertilizer	×	✓	×	×	×	×
[27]	Nitrogen	×	✓	×	×	×	×
[28]	Bell paper	800	✓	0.004	0.005	201.70	-200909
[29]	Lentil	29746.50	✓	0.902	0.06	16.29	-3223.60
[30]	Wheat	60.13	×	3.85	98.6	10.76	44.52
[12]	Bioethanol	×	✓	×	×	×	×
[31]	Tomato	233.2	×	0.01	0.01	×	-21,599.5
[32]	Primrose	×	✓	×	×	×	×
[33]	Tomato puree	×	✓	×	×	×	×
[34]	Apple	83883.37	×	0.72	0.30	3.33	-32621.32

In recent years, the production of Potassium Chloride (KCL) and Potassium Sulfate (K_2_SO_4_) has emerged as the dominant source of potash fertilizers in Iran. However, there is currently limited understanding of the environmental consequences associated with potash production and the potential for mitigation. Furthermore, no studies have yet explored the energy usage and LCA of potash fertilizer production in Iran. So, the objective of this research is to examine the energy utilization and evaluate the environmental impacts of KCL and K_2_SO_4_ production. Moreover, this study aims to identify and propose guidelines for enhancing energy efficiency and reducing the environmental footprint of potash fertilizer production. By identifying key environmental impacts and potential areas for intervention, this research will support decision-makers and industry stakeholders in formulating strategies to enhance energy efficiency, diminish greenhouse gas emissions, and minimize other detrimental environmental impacts associated with potash fertilizer production in Iran. Consequently, the outcomes of this study have the potential to bridge the existing knowledge gap concerning the environmental impacts of potash fertilizer production in Iran and facilitate the development of sustainable production practices and policies. Specifically, conducting an LCA study on KCL and K_2_SO_4_ production will provide valuable insights that can inform initiatives targeting environmental impact reduction and enhanced energy efficiency within the potash fertilizer industry in Iran. In section 2 (Methodology), study area, data collection, energy evaluation and LCA method were discussed. Section 3 (Results and discussion) reports the scientific results, discussion and compare the findings with other similar studies. Also this section include some suggestions. The final part of the research paper will present the conclusions and recommendations of the study. The findings of the study will be discussed in detail, and suggestions for future research will be provided.

## 2 Methodology

### 2.1 Process description

The research commenced on March 1, 2021, with the distribution of questionnaires and concluded on May 25 of the same year. Data analysis and report finalization were completed by December 20, 2023. Data collection was conducted through face-to-face interviews at two potash production facilities. This study involved gathering data directly from employees at the two potash plants, complemented by information extracted from existing literatures. Informed consent from participants was obtained prior to conducting the interviews. Employees were provided with detailed information about the study’s purpose, procedures, and their rights as participants. They were assured of the confidentiality of their responses and given the option to withdraw from the study at any time without any consequences. Consent forms were signed by all participants, ensuring their voluntary participation and understanding of the study. The research did not involve minors or clinical trials. The KCL production plant is located in Khur, Esfehan province, in the central region of Iran, and produces 50,000 tons of KCL per year. Meanwhile, the K_2_SO_4_ production plant is located in Tehran province and produces 20,000 tons of K_2_SO_4_ per year. These two plants are the only potash fertilizer production facilities in Iran.

#### 2.1.1 Potassium chloride production

**Fig 1:**
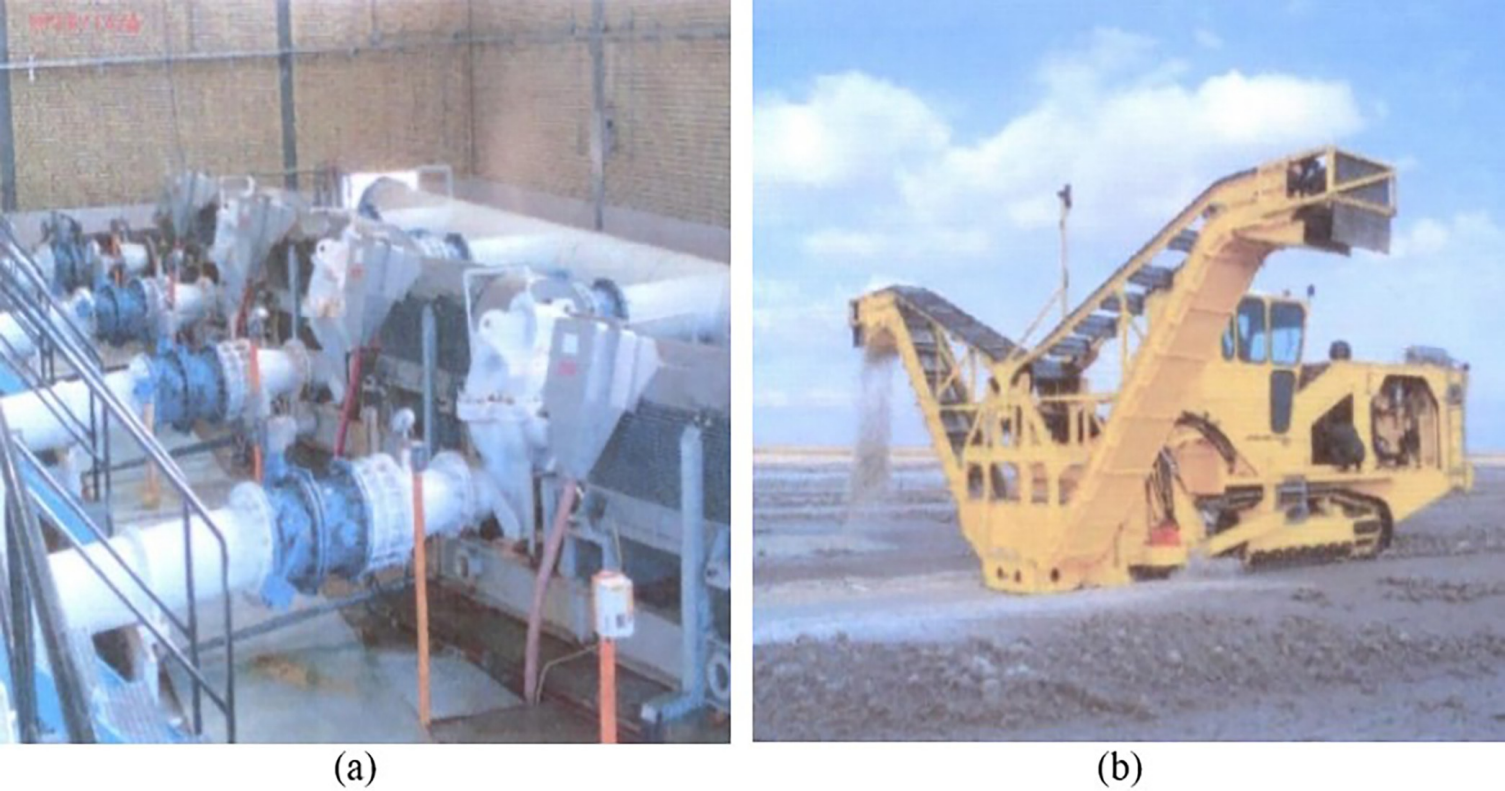
Primary pumping station (a) and mechanical harvester (b) in KCL production process.

The production process of KCL can be divided into seven steps. First, the brine is pumped from Khur lakes through a distance of 9 km using 12 electro pumps. The primary pumping station has four floating turbine pumps and the basic pumping station has eight centrifugal pumps. Then in second step, the pumped brine, with a density of 1.34 kg^-3^, is maintained in six 3,000,000 m^3^ carnallite pools to reach density of 1.45 kgm^-3^ and to allow carnallite to settle. The remaining brine is then pumped back to the playa lakes. In third step, the harvested carnallite is washed and screened before the flotation process in which salts, especially sodium chloride (NaCl) are separated from carnallite. In fourth step, the carnallite is delivered to the decomposition reactor to separate potassium chloride (KCL) from magnesium chloride (MgCl_2_). In this step, water is added to the carnallite, and with the rotation of the decomposition reactor, MgCl_2_ dissolves in water while KCL is separated from the top and bottom of the reactor. Finally, the wet KCL is dried using artificial drying. **Fig 1** shows the primary pumping station (a) and the mechanical harvester (b) used in the KCL production process.

#### 2.1.2 Potassium sulfate production

Potassium Sulfate (K_2_SO_4_) production is based on the reactions of KCL with either sulfates or sulfuric acid [35]. In the K_2_SO_4_ production plant, K_2_SO_4_ is produced through chemical reactions between KCL and sulfuric acid. It is should mentioned that these chemical reactions produce hydrochloric acid as a byproduct along with K_2_SO_4_. These chemical reactions can be:

$$KCl+H_{2}SO_{4}\to KHSO_{4}+HCl$$

$$KCl+KHSO_{4}\to K_{2}SO_{4}+HCl$$ (1)

**Fig 2:**
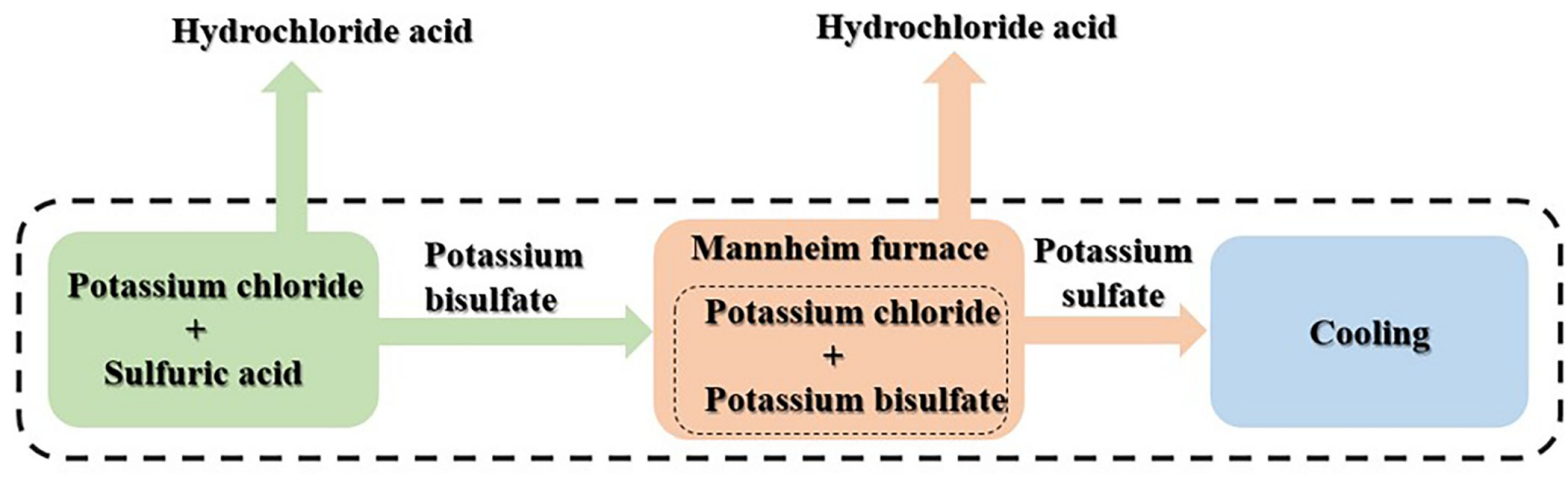
Simplified flowchart of K_2_SO_4_ production process.

The second reaction is endothermic and requires a temperature of approximately 550°C to occur. These chemical reactions were conducted in a Mannheim furnace, which had a diameter of 6 m and a height of 4.5 m. The Mannheim furnace was capable of producing 1.15 tons of K_2_SO_4_ and 0.426 tons of hydrochloric acid per hour. Following the Mannheim process, the K_2_SO_4_ was cooled in a cooling drum. This production plant had two Mannheim furnaces that were in operation continuously. A flowchart of the K_2_SO_4_ production process is presented in **Fig 2**.

### 2.2 Energy use in KCL and K_2_SO_4_ production

**Fig 3:**
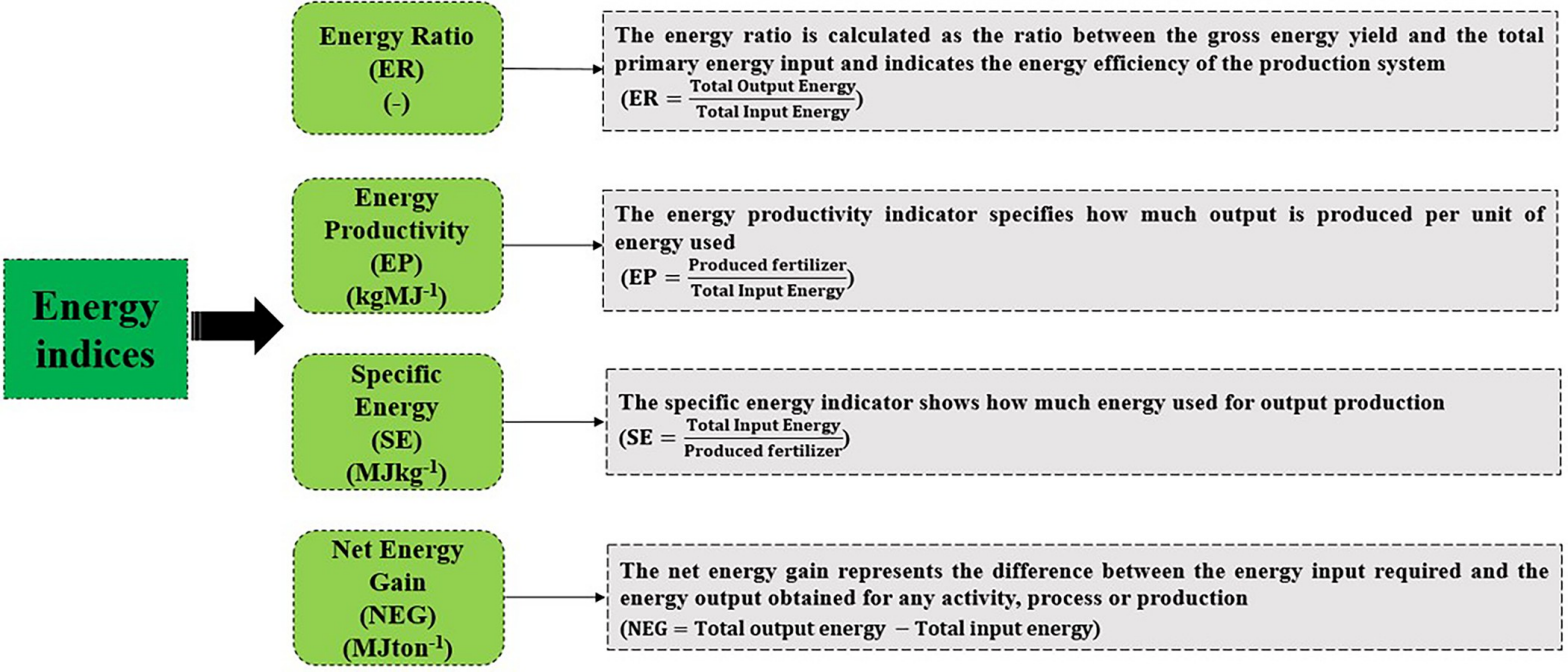
Energy indices in this study [43, 44].

The energy inputs for KCL production include electricity, fuel oil, diesel fuel, human labor, and transportation. Inputs for K_2_SO_4_ production include natural gas, KCL, sulfuric acid, electricity, fuel oil, diesel fuel, human labor, and transportation. The energy conversion factors for each input are presented in **Table 2**. Sulfuric acid is often considered a waste product in the chemical industry [36, 37] and the energy conversion factor for sulfuric acid was assumed to be zero [17]. In K_2_SO_4_ production, 30% hydrochloric acid is produced as a byproduct. The allocation of inputs between K_2_SO_4_ and hydrochloric acid was based on economic allocation, where the economic value of hydrochloric acid was determined to be approximately 10% of the total economic value of the outputs. To calculate energy indices such as Energy Ratio (ER), Energy Productivity (EP), Specific Energy (SE), and Net Energy Gain (NEG), data on energy inputs and outputs were entered into Excel spreadsheets. The energy equivalents of the inputs and outputs were used to perform these calculations, as outlined in the equations presented in **Fig 3**.

**Table 2:** Energy conversion factors of the inputs in production process of KCL and K_2_SO_4_.

Inputs	Energy conversion factor (MJ unit^-1^)	References
Human labor^a^ (h)	1.96	[38, 39]
Electricity^a^ (kWh)	12	[40]
Fuel oil^a^ (L)	47.8	[41]
Natural gas^b^ (m^3^)	49.5	[42]
Diesel fuel^a^ (L)	56.31	[13]
Potassium chloride^b^ (kg)	3.46	Obtained from this study
Sulfuric acid^b^ (kg)	0	[17]
Potassium oxide (K_2_O)	6.4	[40]

^a^ The inputs which are used in both KCL and K_2_SO_4_ production.

^b^ The inputs which are used only in K_2_SO_4_ production.

### 2.3 Life Cycle Assessment

Life Cycle Assessment (LCA) is a technique used to quantify the environmental impacts of products, services, and processes. Standards related to LCA are outlined in ISO 14040 and 14044 [45, 46]. An LCA consists of four main stages, as defined by these standards: goal and scope definition, inventory analysis, impact assessment, and interpretation.

#### 2.3.1 Goal and scope definition

**Fig 4:**
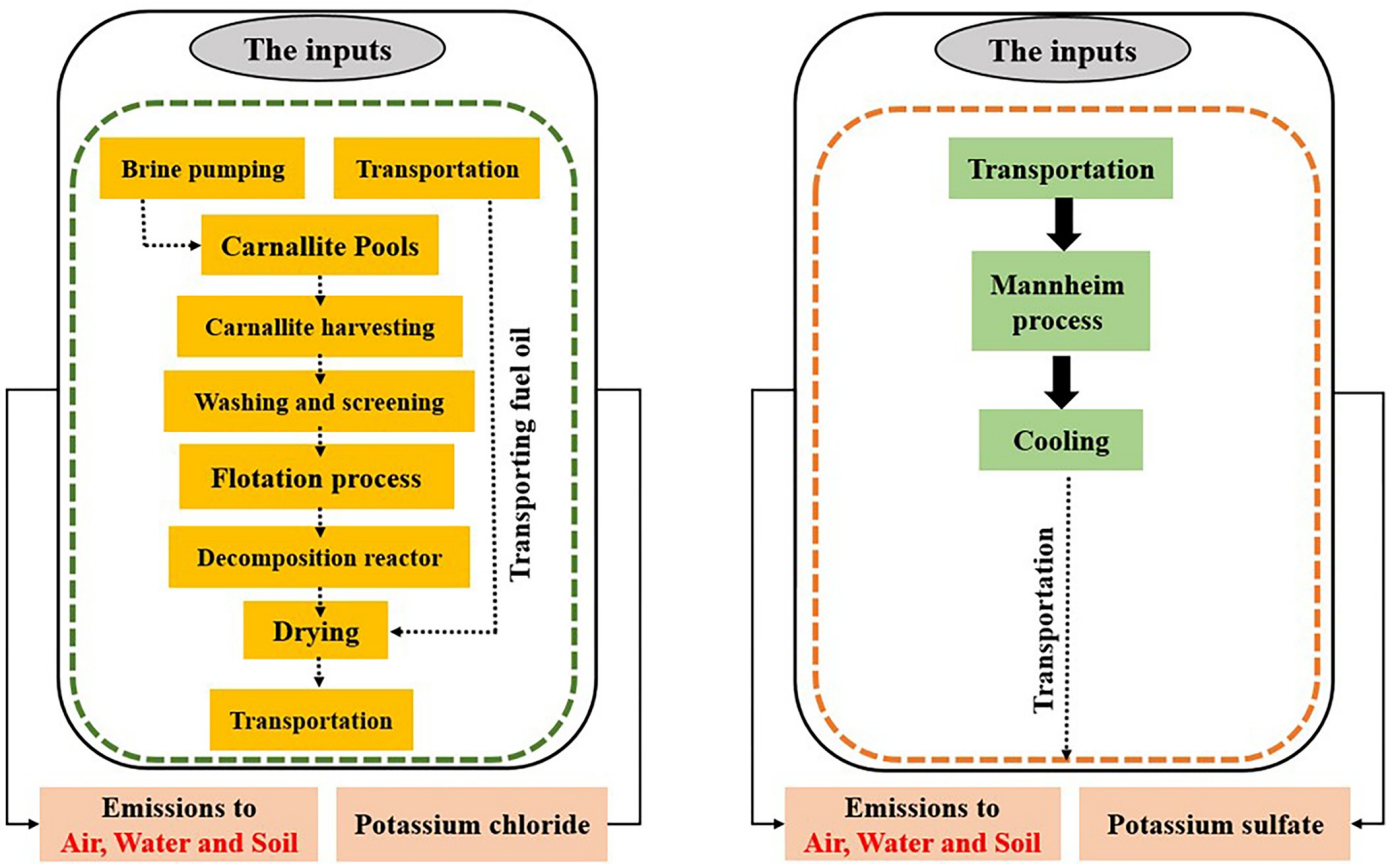
System boundaries of KCL and K_2_SO_4_ production.

The goal and scope definition stage of LCA is to define the aim of the study, the system boundaries, the products, and any assumptions made during the study. The aim of this study was to investigate the environmental impacts of KCL and K_2_SO_4_ production. The system boundaries encompassed the input materials, transportation of inputs and products, and direct emissions from industrial processes (the cradle to the gate) [39]. The system boundaries of the study are illustrated in **Fig 4**.

#### 2.3.2 Functional unit (FU)

In the present study, 1 ton of Potassium Oxide (K_2_O) was considered as the Functional Unit (FU). KCL and K_2_SO_4_contain 60% and 50% K_2_O, respectively. This means that all calculations were based on the amount of inputs required to produce 1.66 tons of KCL and 2 tons of K_2_SO_4_.

#### 2.3.3 Life cycle inventory (LCI)

The second step of LCA is the Life Cycle Inventory (LCI), where an inventory of inputs and outputs is created to meet the aim of the study. The primary data for KCL and K_2_SO_4_ production are presented in **Table 3**. The quantities of inputs for K_2_SO_4_ production were calculated by subtracting the inputs allocated to hydrochloric acid (which was determined to be 10% of the total inputs) from the total inputs. It was assumed that the electricity was generated from natural gas and hydropower (wind power and solar plants were negligible). Also, machineries production and transportation of the labors to the plants were excluded from calculations. In this section, the raw materials, energy flows and transportation considered in this study are elaborated and detailed as follows:

- **Fuel oil:** There are several types of fuel oil with varying specifications. In this study, fuel oil 380 CST was used, which is a heavy, low-quality fuel oil. The density, kinematic viscosity, pour point, flash point, total sulfur, and ash content of fuel oil 380 CST are 990 kgm^-3^, 380 CST, 32°C, 65°C, 3.5 wt%, and 0.15 wt%, respectively. Fuel oil was consumed in the artificial drying step of KCL production and in the Mannheim process of K_2_SO_4_ production.
- **Electricity:** In KCL production, electricity was used for electro pumps, equipment, and lighting. Electricity use in K_2_SO_4_ production was lower than KCL production and only included the use of electrical equipment and lighting.
- **Natural gas:** As an alternative fuel, natural gas was sometimes used instead of fuel oil in the Mannheim process of K_2_SO_4_ production. Although the amount of natural gas used in the Mannheim process was negligible, it was still considered in the calculations.
- **Diesel fuel:** In both plants, diesel fuel was used for industrial machinery and transportation within the plants.
- **Sulfuric acid:** Sulfuric acid produced from the sulfur recovery process was used as a raw material in K_2_SO_4_ production.

**Table 3:** The primary data for one tone K_2_O.

Inputs	KCL: quantity per unit	K_2_SO_4_: quantity per unit
Human labor (h)	8.66	26
Electricity (kWh)	312.5	120
Fuel oil (L)	36	126
Natural gas (m^3^)	-	3
Diesel fuel (L)	5	10.8
Potassium chloride (kg)	-	1660
Transport (tonkm^-1^)	430	550
Sulfuric acid (kg)	-	1000

Direct emission related to the burning of diesel fuel, natural gas, and fuel oil were obtained from EPA 1993, and EPA 1998 [47, 48], respectively. The emission factors for 1 MJ of energy consumption from diesel fuel, fuel oil, and natural gas are presented in **Table 4**.

**Table 4:** Emission factors for 1 MJ energy production from diesel fuel [47, 48].

Emission	Amount (gMJ^-1^ diesel)	Amount (gMJ^-1^ fuel oil)	Amount (gMJ^-1^ natural gas)
Carbon Dioxide (CO_2_)	74.5	86.8	38.7
Sulfur Dioxide (SO_2_)	2.41E-02	3.6	1.93E-04
Sulfur Trioxide (SO_3_)	-	4.8E-02	-
Methane (CH_4_)	3.08E-03	6.8E-03	7.4E-04
Benzene	1.74E-04	-	-
Lead	-	3.68E-05	1.61E-07
Cadmium (Cd)	2.39E-07	9.7E-06	-
Chromium (Cr)	1.19E-06	-	-
Copper (Cu)	4.06E-05	4.28E-05	-
Dinitrogen Monoxide (N_2_O)	2.86E-03	6.33E-03	7.11E-04
Nickel (Ni)	1.67E-06	2.05E-03	6.78E-07
Zinc (Zn)	2.39E-05	7.09E-02	9.37E-06
Benzo(a) pyrene	7.16E-07	-	3.87E-09
Ammonia (NH_3_)	4.77E-04	-	-
Selenium (Se)	2.39E-07	1.66E-04	7.75E-09
Organic Compound	-	2.5E-02	3.5E-03
Volatile Organic Compound (VOC)	-	-	1.7E-03
PAH (Poly Cyclic Hydrocarbons)	7.85E-05	-	-
Hydro Carbons (HC, as NMVOC)	6.80E-02	-	-
Nitrogen Oxides (NOx)	1.06	4.8E-01	-
Carbon Monoxide (CO)	1.50E-01	8.46	-
Particulates (<2.5 μm)	1.07E-01	1.7E-01	2.45E-03

Inventory data for the production of electricity (from natural gas and hydropower), diesel fuel, natural gas, fuel oil, and sulfuric acid were obtained from the Ecoinvent, BUWAL 250, and LCA Food DK databases within the Simapro 8.03.14 software database. Inventory data for KCL, which was used as a raw material in K_2_SO_4_ production, was obtained from this study.

#### 2.3.4 Life Cycle Impact Assessment (LCIA)

The final step of LCA is LCIA, which aims to describe and classify the environmental impacts. There are four steps involved in LCIA: 1) selection of impact categories and classification; 2) characterization; 3) normalization; and 4) weighting.

In this study, the CML-IA baseline method was selected as the LCIA method [33]. The impact categories based on the CML-IA baseline method are Abiotic Depletion (AD), Acidification (AC), Eutrophication (EP), Global Warming (GW), Ozone Layer Depletion (OLD), Human Toxicity (HT), Fresh water aquatic Ecotoxicity (FE), Marine aquatic Ecotoxicity (ME), Terrestrial Ecotoxicity (TE), and Photochemical Oxidation (PO). The Simapro 8.03.14 software, a common software for LCA, was used to assess the environmental impacts of KCL and K_2_SO_4_ throughout their production phase.

## 3 Results and discussion

### 3.1 Analysis of energy use in potassium chloride and potassium sulfate production

**Table 5** presents the average energy consumption for the production of one ton of K_2_O in KCL and K_2_SO_4_ production as 7080.832 and 15691.5 MJ, respectively. The lowest and highest energy inputs in KCL production were human labor and electricity consumption, which totaled 16.97 and 3750 MJ, respectively, accounting for 0.23% and 52.96% of the total energy consumption. The pumping stage was the most energy-intensive process in KCL production, with the pumps consuming significant amounts of electricity to transfer the brine from the playa lakes to the carnallite pools, a distance of approximately 9 km. Fuel oil consumption in the artificial drying step was the second most energy-intensive input in KCL production, accounting for 1720.80 MJ and 24.3% of the total energy consumption. In K_2_SO_4_ production, the lowest and highest energy inputs were for human labor and fuel oil consumption, which totaled 50.96 and 6022.80 MJ, respectively, accounting for 0.32% and 38.39% of the total energy consumption. Fuel oil was used for heating in the Mannheim process, while KCL as a raw material was the second most energy-intensive input, consuming 5743.6 MJ and accounting for 36.60% of the total energy consumption. In both KCL and K_2_SO_4_ production, diesel fuel was used for industrial machinery and transportation of the inputs within the plants. In K_2_SO_4_ production, transportation was responsible for higher energy compared to KCL production, as transporting one ton of K_2_O as K_2_SO_4_ (50% K_2_O) required the transportation of a higher amount of K_2_SO_4_ (2 tons) compared to KCL (1.66 tons), which contains 60% K_2_O. The energy conversion factor of sulfuric acid was considered as 0, and so, this input had no share in the total energy consumption.

**Table 5:** Energy equivalents and the share of the inputs and the total energy equivalent for one ton K_2_O.

Inputs-Output	Potassium chloride: Energy equivalent (MJ)	Potassium chloride: Percentage	Potassium sulfate: Energy equivalent (MJ)	Potassium sulfate: Percentage
Human labor (h)	16.97	0.23	50.96	0.32
Electricity (kWh)	3750	52.96	1440	9.17
Fuel oil (L)	1720.8	24.30	6022.8	38.39
Natural gas (m^3^)	-	-	148.5	0.94
Diesel fuel (L)	281.55	3.97	608.14	3.87
Potassium chloride (kg)	-	-	5743.6	36.60
Transport (Ton.km)	1311.5	18.53	1677.5	10.69
Total energy input (MJ)	7080.82	100	15691.75	100
Output				
Potassium oxide (K_2_O)	6400	-	6400	-

In summary, the production of K_2_SO_4_ demands more energy than KCL due to the complexity of the synthesis processes, the purity of raw materials, the chemical properties of the reactions involved, and the energy-intensive steps required for product separation and purification. The results presented in **Table 5** show that electricity accounts for more than half of the total energy used in KCL production. The KCL plant is located in the Khur Playa, which is a vast desert in Iran with high potential for solar energy production. Therefore, installing a photovoltaic power station near the plant would significantly reduce the total energy input in KCL production. In K_2_SO_4_ production, fuel oil consumption accounted for 38.39% of the total input energy used in the production process. However, compared to other fossil fuels, fuel oil is not an efficient or eco-friendly fuel [49]. The only reason to utilize fuel oil is its cheap price compared to other fossil fuels. Despite the fact that fuel oil produces more heat per BTU, gas is generally considered more efficient [50]. To improve energy efficiency and reduce environmental impacts, the government could allocate subsidies for alternative energy use or introduce new legislation to encourage the use of more efficient and eco-friendly fuels in fertilizers production.

Although there have been a few studies on energy use of fertilizers production in Iran [12, 17] no studies have been published on energy use of potash fertilizer production in the country. Farahani et al. (2017) [12] reported that the total energy consumption in the granulation process of NPK-15:8:15 was 1659.92 MJ per ton of fertilizer, with electricity consumption being the highest at 1527.96 MJ. Salami et al. 2010 estimated the total energy equivalent for producing each kg of single superphosphate to be 3.5 MJ. They also mentioned that with an energy equivalent sulfuric acid value of -2.2, the total required energy was 2.07 MJ. The single superphosphate produced in their study contained 22% phosphate, and dividing 3.5 MJ per 22% resulted in a total energy input of 15.90 MJ per kg of phosphate. Based on the results of these studies, it can be concluded that potash fertilizers produced in Iran, whether as KCL or K_2_SO_4_, are less energy-intensive than phosphate fertilizers.

**Table 6** shows the energy ratio, energy productivity, specific energy, and net gain energy for H_2_SO_4_ and KCL. The ER for K_2_SO_4_ was 0.40, EP was 0.06 kgMJ^-1^, SE was 15.6 MJkg^-1^ and NEG calculated -680.82 MJton^-1^. On the other hand, all the above indices for KCL was 0.90, 0.14 kgMJ^-1^, 7.08 MJkg^-1^, and -9291.75 MJton^-1^, respectively. Given the percentage of K_2_O, the energy equivalents of K_2_SO_4_ and KCL were calculated as 7.8 MJkg^-1^ and 4.24 MJkg^-1^, respectively. Skowrońska and Filipek (2014) [51], conducted a review study on mineral fertilizer production and reported that the energy equivalent of KCL based on European average data was 10.06 MJkg^-1^ K or 8.38 MJkg^-1^ K_2_O. Gellings and Parmenter (2004) [52], also reported that energy required for production, packaging and transportation of potash fertilizers (world average) is 12.8 MJkg^-1^ K_2_O.The difference between their study and the present study can be attributed to factors such as different packaging materials, transportation distance, and the year of the studies conducted. He et al. (2012) [53] reported that the energy ratio in steel production increased from 0.536 in 2001 to 0.600 in 2006 and to 0.614 in 2008. Ramírez and Worrel (2006) [54], reported that energy required to produce 1 kg potash KCL from about 7 MJkg^-1^ in 1961 decreased to about 5 MJkg^-1^ in 2001. This demonstrates the continuous potential for improvement in industries through advancements in science and technology.

**Table 6:** Energy indices in H_2_SO_4_ and KCL production.

Item	Unit	K_2_SO_4_	KCL
Energy Ratio (ER)	-	0.40	0.90
Energy Productivity (EP)	kgMJ^-1^	0.06	0.14
Specific Energy (SE)	MJkg^-1^	15.6	7.08
Net Energy Gain (NEG)	MJton^-1^	-680.82	-9291.75

### 3.2 Interpretation of LCA results

**Table 7** presents the impacts assessment of K_2_SO_4_ and KCL production in various categories for one ton of K_2_O production. The results show that the Global Warming Potential (GWP) of K_2_SO_4_ production was higher at 1820.86 kg CO_2_ eq compared to KCL production at 888.33 kg CO_2_ eq. Based on the economic allocation, 10% of the inputs used in K_2_SO_4_ production allocated to hydrochloric acid and excluded from the calculation of the impact categories. As expected, K_2_SO_4_ had significantly higher impacts in all categories compared to KCL. Only in ME category, difference was negligible due to the high amount of electricity used in KCL production. These results suggest that KCL production has lower environmental impacts compared to K_2_SO_4_ production in all categories. This can be attributed to the differences in the production processes and energy inputs used in each process. The high energy consumption in K_2_SO_4_ production, especially fuel oil used in Mannheim process, resulted in the significant environmental impacts in all the categories. So, measures such as using alternative energy sources or improving energy efficiency could help to reduce the environmental impacts related to K_2_SO_4_ production.

**Table 7:** Impact categories for one tone K_2_O.

Impact category	Unit	KCL	K_2_SO_4_
Abiotic Depletion (AD)	kg Sb eq	6.97	13. 8
Acidification (AC)	kg SO_2_ eq	8.16	40.7
Eutrophication (EP)	kg PO_4_^-2^ eq	0.46	1.6
Global Warming Potential (GWP)	kg CO_2_ eq	888.33	1820.86
OzoneL Depletion (OLD)	kg CFC-11 eq	4.86E-05	2.98E-04
Human Toxicity (HT)	kg 1,4-DB eq	279.87	886.18
Fresh water aquatic Ecotoxicity (FE)	kg 1,4-DB eq	64.4	155
Marine aquatic Ecotoxicity (ME)	kg 1,4-DB eq	224963.8	264440.6
Terrestrial Ecotoxicity (TE)	kg 1,4-DB eq	0.406	4.82
Photochemical Oxidation (PO)	kg C_2_H_4_	0.358	1.6

Skowrońska and Filipek (2014) [51], conducted a study on the environmental emissions of mineral fertilizers based on European average data. The amount of CO_2_ eq for KCL production based on K was reported as 0.58 kg CO_2_ eq per one kg of K (0.48 kg CO_2_ eq per one kg of K_2_O), which is lower than the value obtained in this study. The difference is likely due to the type of inputs used and the use of the clean fuels in their study. Hayashi et al. (2014) [55], reported the CO_2_ eq for K_2_SO_4_ production in Europe and Japan. The study showed that the amount of GWP category for K_2_SO_4_ production in Europe (1.36 kg CO_2_ eq/kg K_2_O) was higher than Japan (0.98 kg CO_2_ eq/kg K_2_O). In the present study, the amount of GWP category for K_2_SO_4_ production was even higher compared to both Europe and Japan. These differences in reports can be attributed to the differences in fertilizer production technologies, databases and inputs used in each study. Chen et al. (2018) [23], reported that GW category for 1 ton K_2_O (as KCL) production obtained as 190 kg CO_2_ eq which is significantly lower than 888.33 kg CO_2_ eq obtained in this study. There are many reasons for this difference such as different database, difference in production process, transportation distance and study assumption, but most important reason for this difference could be attributed to using significantly less electricity in their study. In our study, the KCL plant is located at a distance of 9 km from the lakes using 12 electro pumps to transfer brine to the plant. Considering renewable and clean sources to generate electricity like installing a photovoltaic power station near the plant can significantly reduce emissions in KCL production in all categories.

To reduce the environmental impact of potash fertilizer production, it is important to select the best available technology and use clean fuels such as biogas, biodiesel, or electricity generated from renewable energy sources [56, 57]. These measures can significantly reduce the environmental impact of potash fertilizer production.

**Fig 5:**
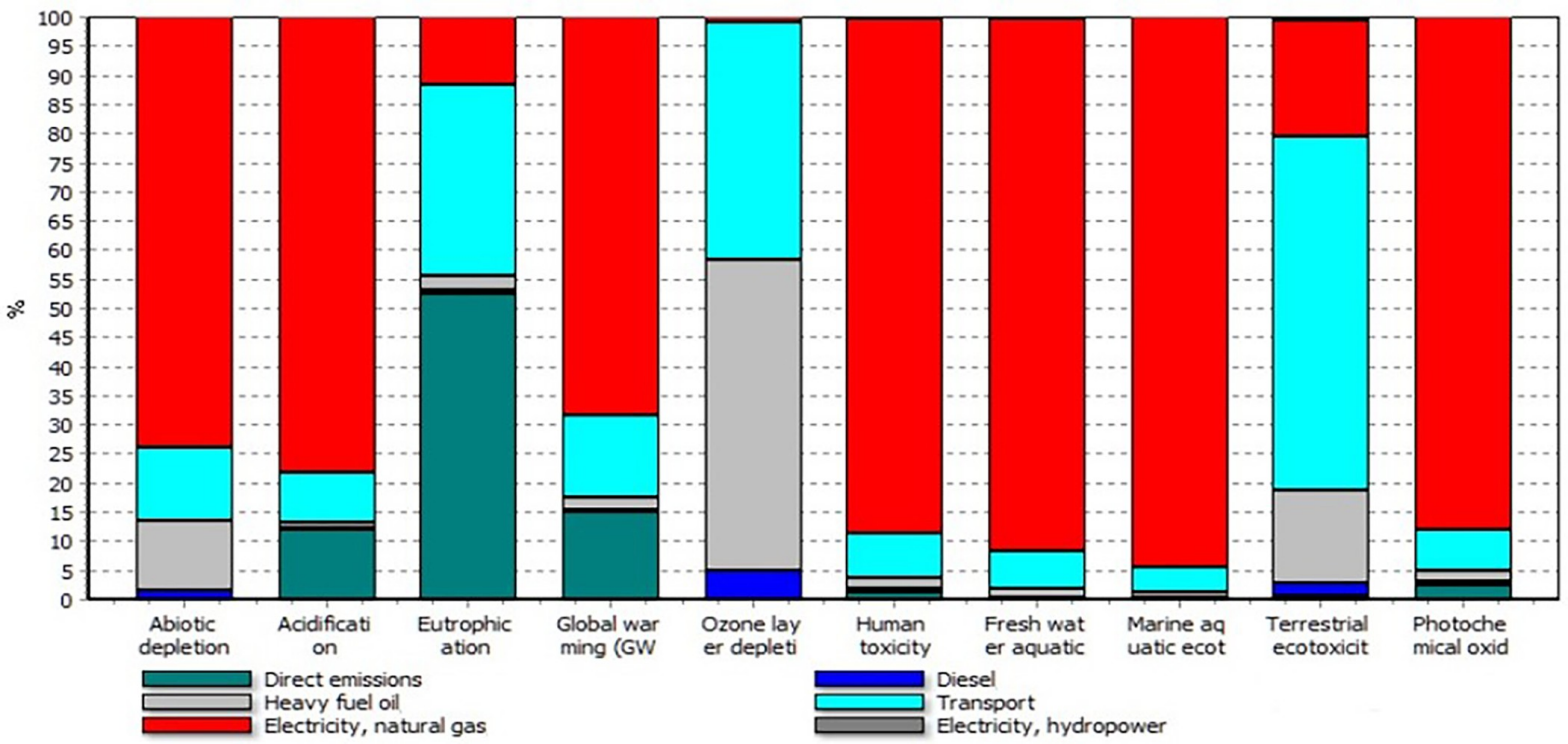
Contribution of inputs and direct emissions to environmental impact categories in KCL production.

**Fig 5** illustrates the contribution of inputs and direct emissions to the impact categories in KCL production. The results show that the high quantity of electricity used in the pumping step was the most significant contributor to the impact categories, except for EP and OEL. Electricity generated from natural gas had the largest contribution to AD, AC, GWP, HT, FE, ME, and PO, as 74.54%, 77.08%, 68.32%, 87.86%, 92.54%, 94.65%, and 86.66%, respectively. Chen et al., (2018) reported that the overall environmental impacts of KCL production in China were mainly caused by electricity, which is similar to the results obtained in this study. Although electricity in their study was significantly lower than this study. EP and OEL were mainly due to direct emissions (53.24%) and fuel oil (52.86%), respectively.

The emissions from electricity are dependent on the type of power plant used for electricity generation. In Iran, electricity is mainly generated from natural gas (94.3%) and hydropower (4.9%) (Farahani et al., 2019). The share of the other power plants such as nuclear and wind is less than 1% of the electricity generated in Iran. In this study, it was assumed that all electricity used in KCL production was generated from natural gas power plants (95.1%) and hydropower plants (4.9%).

**Fig 6:**
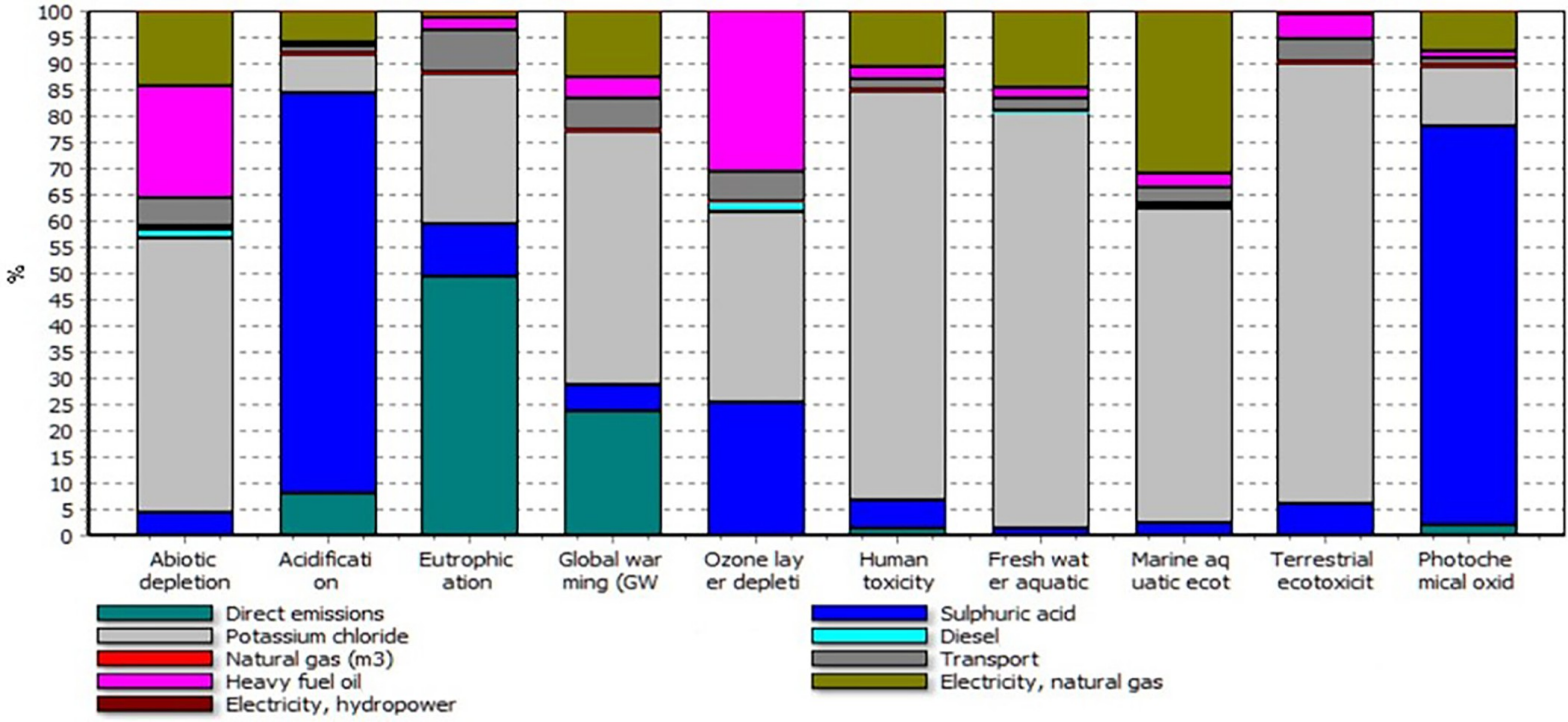
Contribution of inputs and direct emissions to environmental impact categories in K_2_SO_4_ production.

**Fig 6** shows the contribution of inputs and direct emissions to the impact categories in K_2_SO_4_ production. The results indicate that KCL used as a raw material was the most significant contributor to most impact categories. Sulfuric acid was the main contributor in AC and PO, accounting for 72.23% and 75.52%, respectively. EP was mainly due to direct emissions (49.68%). KCL had the largest share in AD, GW, OEL, HT, FE, ME, and TE, accounting for 52.02%, 48.12%, 34.24%, 77.43%, 78.34%, 59.26%, and 83.48%, respectively. KCL was also the second contributor to EP and PO, accounting for 28.35% and 11.14%, respectively.

These results suggest that reducing the environmental impact of K_2_SO_4_ production requires measures to optimize the use of KCL and sulfuric acid, which are the major contributors to most impact categories. This can be achieved by improving the efficiency of the production process, using alternative raw materials, and reducing emissions from sulfuric acid. Additionally, reducing direct emissions from the production process can also help to reduce the impact of K_2_SO_4_ production on the environment.

**Table 8** presents the direct emissions values (kg) into the air in KCL and K_2_SO_4_ production. The direct emissions from diesel fuel, fuel oil, and natural gas combustion were calculated based on **Table 3**. The fuel oil used for the artificial drying step and Mannheim process had the highest effect on all types of direct emissions in both KCL and K_2_SO_4_ production. The fuel oil and diesel fuel used for transportation had the highest effect on CO_2_, Sulfur Dioxide (SO_2_), cadmium, chromium, and other emissions. As shown in **Figs 5 and 6**, direct emissions were the highest contributor to EP and the second-highest contributor to GW in both KCL and K_2_SO_4_ production. In KCL production, about 16% of GW was due to direct emissions, while in K_2_SO_4_ production, direct emissions had a contribution of 24% to GW. In KCL production, however, direct emissions had a higher contribution to AC and EP.

**Table 8:** Direct emissions values (kg) into air in KCL and K_2_SO_4_ production.

Emission	KCL	H_2_SO_4_
Carbon Dioxide (CO_2_)	173.15	583.67
Sulfur Dioxide (SO_2_)	6.31	22.11
Sulfur Trioxide (SO_3_)	0.084	0.29
Methane (CH_4_)	0.012	0.043
Benzene	4.9E-05	1.06E-04
Lead	6.45E-05	2.26E-04
Cadmium (Cd)	1.71E-05	5.97E-05
Chromium (Cr)	3.35E-07	7.24E-07
Copper (Cu)	8.65E-05	2.87E-04
Dinitrogen Monoxide (N_2_O)	0.011	0.04
Nickel (Ni)	0.0035	0.012
Zinc (Zn)	0.12	0.43
Benzo(a)pyrene	2.02E-07	4.36E-07
Ammonia (NH_3_)	1.34E-04	2.9E-04
Selenium (Se)	2.91E-04	1.01E-03
Organic compound	0.043	0.15
Volatile Organic Compound (VOC)	-	2.52E-04
PAH (poly cyclic hydrocarbons)	2.21E-05	4.77E-05
Hydro Carbons (HC, as NMVOC)	0.019	0.041
Nitrogen Oxides (NOx)	1.13	3.59
Carbon Monoxide (CO)	14.87	52.01
Particulates (<2.5 μm)	0.328	1.11

The results shows that fuel oil has a higher emission factor compared to diesel and natural gas for 1 MJ of production. However, fuel oil is often used in the Mannheim process and artificial drying in potash fertilizer production due to its cheaper price compare to natural gas. To reduce direct emissions in KCL and K_2_SO_4_ production, natural gas can be used instead of fuel oil. **Table 9** illustrates the impact of replacing fuel oil with natural gas on the impact categories in both KCL and K_2_SO_4_ production. The results show that replacing fuel oil with natural gas reduced all impact categories except FE and ME. The highest reduction was observed in OEL, with a reduction of 52.7% and 30.4% in KCL and K_2_SO_4_ production, respectively. GW also reduced by 7.3% and 14.7% in KCL and K_2_SO_4_ production, respectively.

**Table 9:** The impact of replacing fuel oil with natural gas on the categories in both KCL and K_2_SO_4_ production.

	K_2_SO_4_		KCL	
Impact category	Fuel oil	Natural gas	Fuel oil	Natural gas
Abiotic Depletion (AD)	13. 8	13.30 (-3.6%)	6.97	6.83 (-2.2%)
Acidification (AC)	40.7	39.07 (-4.1%)	8.16	7.75 (-5.1%)
Eutrophication (EP)	1.6	1.16 (-27.2%)	0.46	0.36 (-21%)
Global Warming Potential (GWP)	1820.86	1565.94 (-14%)	888.33	817.26 (-8.2%)
Ozone Layer Depletion (OLD)	2.98E-04	2.09E-04 (-29%)	4.86E-05	2.04E-05 (-51.8%)
Human Toxicity (HT)	886.18	873.77 (-1.4%)	279.87	277.07 (-1.1%)
Fresh water aquatic Fcotoxicity (FE)	155	158.1 (+2%)	64.4	65.17(+1.2%)
Marine aquatic Ecotoxicity (ME)	264440.6	277662.6 (+5%)	224963.8	276705.47 (+2.3%)
Terrestrial Ecotoxicity (TE)	4.82	4.62 (-4.1%)	0.406	0.361 (-13%)
Photochemical Pxidation (PO)	1.6	1.57 (-1.7%)	0.358	0.349 (-2.1%)

Kongshaug 1998 [58], reported that 1.2% of global GHG emissions are attributed to greenhouse gases emitted from fertilizer production. In both KCL and K_2_SO_4_ plants, direct emissions from fossil fuel combustion were the second-highest contributor to GWP, accounting for 13.02% and 25.17%, respectively. To reduce direct emissions in KCL and K_2_SO_4_ plants, fossil fuels can be replaced with clean energies such as biogas and biodiesel. Additionally, reducing transportation distances for inputs and increasing energy efficiency can improve energy consumption and reduce environmental impacts. The government can encourage producers to use natural gas or other clean fuels instead of fuel oil by providing subsidies for natural gas and other clean fuels. Rules can also be set to use GHG absorption systems in Mannheim process (in K_2_SO_4_ production) and artificial dryers (in KCL production) to reduce GHG emissions.

As the results indicate, electricity dominates the eight impact categories in KCL production. Considering the vast vacant and unimpeded space in the great desert of Iran where the KCL plant is located, installing a photovoltaic power station near the plant can significantly reduce emissions in KCL production in all categories. In Iran, 93% of electricity is generated from fossil fuels in thermal power plants [33], so it is understandable that electricity causes significant emissions in the categories. Therefore, installing a photovoltaic power station is strongly recommended. The Environment Department and Ministry of Industry and Trade and Mines of Iran can obligate the plant to install a photovoltaic power station or consider allocating a budget for this purpose after further studies.

It is suggested that a consequential LCA (CLCA) study conducted before final decisions about any change. CLCA aiming to describe how environmental burdens will change in response to possible decisions [59]. CLCA method can include both increase and reduction in the environmental impacts affected by decision. However, CLCA requires databases which included needed marginal data. It is expected that CLCA become easier to conduct if future database include more of marginal data [59].

## 4 Conclusion

The aim of this study was to estimate the energy consumption and environmental impacts of producing one ton of potassium oxide (K_2_O) in KCL and K_2_SO_4_ production, identify hotspots, and propose potential ways to reduce environmental impacts. The results showed that the energy equivalents of 1 kg of K_2_O as K_2_SO_4_ and KCL were calculated as 15.6 MJkg^-1^ and 7.08 MJkg^-1^, respectively. Given that energy required to produce 1 kg potash decreased through the years, the continuous studies to identify potential improvements in industries through advancements in science and technology is essential. To improve energy efficiency and environmental performance, using clean energy sources instead of fossil fuels, reducing transportation distances, and increasing energy efficiency of the processes are suggested. Additionally, GHG absorption systems can be installed in the Mannheim process for K_2_SO_4_ production and the artificial dryer for KCL production to reduce GHG emissions. The results indicated that replacing fuel oil with natural gas reduced all impact categories except FE and ME. However, fuel oil is preferred by producers due to its lower costs. So, it is recommend government intervention, such as banning the use of fuel oil or providing subsidies for clean fuels like natural gas. Electricity had the largest contribution to the eight impact categories in KCL production. Also, electricity used in KCL production in this study was approximately 3 times higher than similar study due to the distance between the lakes and the plant. So, installing a photovoltaic power station near the KCL plant, which has vast vacant and unimpeded space, can improve energy efficiency and reduce emissions in all categories. The government could oblige the plant to install a photovoltaic power station or allocate a budget for this purpose after further feasibility and economic studies. Governments, policymakers, and industry stakeholders should consider implementing the proposed solutions to achieve sustainable production and minimize environmental harm.

## Supporting information

S1 Data PCH. (XLS)

S2 Data PS. (XLS)

S1 Nomenclature (DOCX)

## References

[1] IPCC. (2014) Climate Change 2014: Mitigation of climate change. Contribution of Working groups III to the Fifth Assessment Report of the Intergovernmental Panel on Climate Change. Cambridge University Press, Cambridge, United Kingdom and New York, USA.

[2] Qiu S., Yang H., Zhang S., Huang S., Zhao S., Xu X., et al. (2023). Carbon storage in an arable soil combining field measurements, aggregate turnover modeling and climate scenarios. CATENA, 220, 106708. 10.1016/j.catena.2022.106708

[3] Shang M., & Luo J. (2021). The Tapio Decoupling Principle and Key Strategies for Changing Factors of Chinese Urban Carbon Footprint Based on Cloud Computing. International Journal of Environmental Research and Public Health, 18(4), 2101. doi: 10.3390/ijerph18042101 33670040 PMC7926756

[4] Yu W., Hayat K., Ma J., Fan X., Yang Y., Zhang Z., et al. (2024). Effect of antibiotic perturbation on nitrous oxide emissions: An in-depth analysis. Critical Reviews in Environmental Science and Technology, 1–21. 10.1080/10643389.2024.2339795

[5] Zhao Y., Wang J., Cao G., Yuan Y., Yao X., & Qi L. (2023). Intelligent control of multilegged robot smooth motion: a review. IEEE Access.

[6] Cheng K., Pan G.X., Smith P., Luo T., Li L.Q., Zheng J.W., et al. (2011). Carbon footprint of China’s crop production—An estimation using agro-statistics data over 1993–2007. Agr. Ecosyst. Environ. 142, 231–237.

[7] Guo J., Liu Y., Zou Q., Ye L., Zhu S., & Zhang H. (2023). Study on optimization and combination strategy of multiple daily runoff prediction models coupled with physical mechanism and LSTM. Journal of Hydrology, 624, 129969.

[8] Ouikhalfan M., Lakbita O., Delhali A., Assen A.H., & Belmabkhout Y. (2022). Toward net-zero emission fertilizers industry: greenhouse gas emission analyses and decarbonization solutions. Energy Fuels. 36(8): 4198–4223.

[9] Liu Z., Xu Z., Zheng X., Zhao Y., & Wang J. (2024). 3D path planning in threat environment based on fuzzy logic. Journal of Intelligent & Fuzzy Systems, (Preprint), 1–14.

[10] Rawashdeh R., & Mazwell P. (2014) Analyzing the world potash industry. Resource Policy. 41:143–151.

[11] Zhu C., Li X., Wang C., Zhang B., & Li B. (2024). Deep Learning-Based Coseismic Deformation Estimation from InSAR Interferograms. IEEE Transactions on Geoscience and Remote Sensing.

[12] Farahani S.S., & Asoodar M. (2017). Life cycle environmental impacts of bioethanol production from sugarcane molasses in Iran. Environ Sci Pollut Res 24:22547–22556. doi: 10.1007/s11356-017-9909-1 28804804

[13] Farahani S.S., Rajabipour A., & Keyhani A. (2016) Energy use and economic analysis of NPK-15:8:15 fertilizer granulation process in Iran. Journal of the Saudi Society of Agricultural Sciences, 16(3):265–269.

[14] Fadare D.A., Bamiro O.A., & Oni A.O. (2010) Energy and cost analysis of organic fertilizer production in Nigeria. Energy. 35:332–340.

[15] Ahlgren S., Bernessan S., Nordberg A., & Hansson P. (2010) Nitrogen fertilizer production based on biogas-energy input, environmental impact and land use. Bioresource Technology. 701:7181–7184. 10.1016/j.biortech.2010.04.006 20435469

[16] Rafiquel I., Weber C., Lehmann B., & Voss A. (2005) Energy efficiency improvements in ammonia production-perspectives and uncertainties. Energy. 30:2487–2504.

[17] Salami P., Ahmadi H., & Keyhani A. (2010) Estimating the equivalent energy for single super phosphate production in Iran. Journal of Scientific Review. 2: 64–72.

[18] Liang A., Lv T., Pan B., Zhu Z., Haotian R., Xie Y., et al. (2024). Dynamic simulation and experimental studies of molecularly imprinted label-free sensor for determination of milk quality marker. Food Chemistry, 449, 139238. doi: 10.1016/j.foodchem.2024.139238 38583401

[19] Xie G., Fu B., Li H., Du W., Zhong Y., Wang L., et al. (2024). A gradient-enhanced physics-informed neural networks method for the wave equation. Engineering Analysis with Boundary Elements, 166, 105802.

[20] Ding B., Zhang J., Zheng P., Li Z., Wang Y., Jia G., et al. (2024). Water security assessment for effective water resource management based on multi-temporal blue and green water footprints. Journal of Hydrology, 632, 130761.

[21] Manjare S., & Mohite R. (2012) Application life cycle assessment to diammonium phosphate production. Advanced Materials Research. Vols. 354–355, pp. 256–265.

[22] Nemecek T., Kägi T., & Blaser S. (2007). Life Cycle Inventories of Agricultural Production Systems. Final Report Ecoinvent v2. 0; No. 15.

[23] Chen W., Geng Y., Hong J., Yang D., & Ma X. (2018) Life cycle assessment of potash fertilizer production in China. Resources, Conservation & Recycling. 138:238–245.

[24] Mousavi M., Taki M., Ghaseminejad Raeini M., & Soheilifard F. (2023). Evaluation of energy consumption and environmental impacts of strawberry production in different greenhouse structures using life cycle assessment (LCA) approach. Energy. 280: 128087.

[25] Payandeh Z., Jahanbakhshi A., Mesri-Gundoshmian T., & Clark S. (2021). Improving energy efficiency of barley production using joint data envelopment analysis (DEA) and life cycle assessment (LCA): evaluation of greenhouse gas emissions and optimization approach. Sustainability. 13(11):6082.

[26] Alengebawy A., Mohamed B., Jin K., Liu T., Ghimire N., Samer M., et al. (2022). A comparative life cycle assessment of biofertilizer production towards sustainable utilization of anaerobic digestate. Sustainable Production and Consumption. 33: 875–889.

[27] Gaidajis G., & Kakanis I. (2021). Life cycle assessment of nitrate and compound fertilizers production- A case study. Sustainability. 13(1): 148.

[28] Naderi S.A., Dehkordi A.L., Taki M. (2019). Energy and environmental evaluation of greenhouse bell pepper production with life cycle assessment approach. Environ Sustain Indicat. 3:100011.

[29] Elhami B., Khanali M., & Akram A. (2017). Combined application of Artificial Neural Networks and life cycle assessment in lentil farming in Iran. Info Process Agric 2017;4(1): 18–32.

[30] Mondani F., Aleagha S., Khoramivafa M., & Ghobadi R. (2017). Evaluation of greenhouse gases emission based on energy consumption in wheat Agroecosystems. Energy Rep. 3:37–45.

[31] Pahlavan R., Omid M., & Akram A. (2011). Energy use efficiency in greenhouse tomato production in Iran. Energy. 36(12):6714–9.

[32] Salehpour T., Khanali M., & Rajabipour A. (2020). Environmental impact assessment for ornamental plant greenhouse: life cycle assessment approach for primrose production. Environ Pollut. 266:115258. doi: 10.1016/j.envpol.2020.115258 32771865

[33] Farahani S.S., Soheilifard F., Ghaseminejad-Raini M., & Kokei D. (2019) Comparison of different tomato puree production phases from an environmental point of view. The International Journal of Life Cycle Assessment. 24(10):1817–1827.

[34] Naderi S., Raini M.G.N., & Taki M. (2020). Measuring the energy and environmental indices for apple (production and storage) by life cycle assessment (case study: semirom county, Isfahan, Iran). Environ Sustain Indicat. 6:100034.

[35] Grzmil B.U., & Kic B. (2005) Single-stage process for manufacturing of potassium sulphate from sodium sulphate. The 32nd International Conference of the Slovak Society of Chemical Engineering, Tatranské Matliare, Poland. 23–27 May.

[36] Worrel E., Van Heijningen R.J.J., De Castro J.F., Hazewinkel J.H.O., De Beer J.G., Faaji A.P.C., et al. (1994) New gross energy-requirement figures for materials production. Energy. 19:627–640.

[37] Li J., Lu T., Yi X., Hao R., Ai Q., Guo Y., et al. (2024a). Concentrated solar power for a reliable expansion of energy systems with high renewable penetration considering seasonal balance. Renewable Energy, 226, 120089.

[38] Ranjbar I., Ajabshirchi Y., Taki M., Ghobadifar A., 2013. Energy consumption and modeling of output energy with MLP Neural Network for dry wheat production in Iran. Elixir Agriculture 62, 17949–17953. 10.1016/j.jssas.2014.05.001

[39] Taki M., Yildizhan H., 2018. Evaluation the sustainable energy applications for fruit and vegetable productions processes; case study: greenhouse cucumber production. J. Clean. Prod. 199, 164e172. 10.1016/j.jclepro.2018.07.136.

[40] Kitani O. (1999) CIGR Handbook of Agricultural Engineering, Energy and Biomass Engineering. ASAE Publication, St Joseph MI, Volume V.

[41] Abdi R., Taki M., Akbarpour M., 2012. An Analysis of Energy input-output and Emissions of Greenhouse Gases from Agricultural Productions. Int. J. Nat. Eng. Sci 6(3), 73–79. 10.1016/j.egyr.2016.05.007

[42] Yildizhan H, Taki M. 2019. Sustainable management and conservation of resources for different wheat production processes; cumulative exergy consumption approach. Int. J. Exergy, 28(4): 404–422. 10.1504/IJEX.2019.099295.

[43] Azizpanah A., Fathi R., & Taki M. (2022). Eco-energy and environmental evaluation of cantaloupe production by life cycle assessment method, Environmental science and pollution research, 1854–1870 (2023). 10.1007/s11356-022-22307-2. PMC9362568 35922594

[44] Taki M., Soheili-Fard F., Rohani A., Chen G. Yildizhan H., 2018. Life cycle assessment to compare the environmental impacts of different wheat production systems. J. Clean. Prod 197, 195–207. 10.1016/j.jclepro.2018.06.173.

[45] ISO 14040. (2006a) Environmental Management: Life Cycle Assessment: Principles and Framework.

[46] ISO 14044. (2006b) Environmental Management: Life Cycle Assessment: Requirment and Guidelines.

[47] EPA-Environmental Protection Agency. (1993) Emission factor documentation for AP-42 Section 1.3-Fuel oil combustion, Technical support division, Office of Air Quality Planning and Standards, Research Triangle Park, NC. ‹https://www3.epa.gov/ttnchie1/old/ap42/ch01/s03/bgdocs/b01s03_nov1996.pdf›.

[48] EPA-Environmental Protection Agency. (1998) Emission factor documentation for AP-42 Section 1.4-Natural gas combustion, Technical support division, Office of Air Quality Planning and Standards, Research Triangle Park, NC. ‹http://www3.epa.gov/ttnchie1/ap42/ch01/bgdocs/b01s04.pdf ›.

[49] Higazy M., Essa K.S.M., Mubarak F., El-Sayad E.M., Sallam A.M., & Talaat S. (2019). Analytical study of fuel switching from heavy fuel oil to natural gas in clay brick factories at arab Abu Saed, Greater Cairo. Scientific Reports. 9: 10081. doi: 10.1038/s41598-019-46587-w 31300745 PMC6626056

[50] Vasudevan S., Farooq S., Karimi I.A., Saeys M., Quah M., & Agrawal R. (2016). Energy penalty estimates for CO2 capture: Comparison between fuel types and capture-combustion modes. Energy. 103: 709–714.

[51] Skowrońska M, Filipek T (2014) Life cycle assessment of fertilizers: a review. Int. Agrophys. 28:101–110.

[52] Gellings C.W., & Paramenter K.E. (2004). Energy efficiency fertilizer production and use. Efficient use and Conservation of Energy. Encyclopedia of Life Support Systems (EOLSS). UNESCO, Eolss Publishers, Oxford, UK.

[53] He F., Zhang Q., Lei J., Fu W., & Xu X. (2012). Energy efficiency and productivity change of China’s iron and steel industry: Accounting for undesirable outputs. Energy Policy. 54: 204–213.

[54] Ramírez C.A., & Worrell E. (2006). Feeding fossil fuels to the soil An analysis of energy embedded and technological learning in the fertilizer industry. Resources, Conservation and Recycling. 46: 75–93.

[55] Hayashi K., Makino N., Shobatake K., & Hokazono S. (2014) Influence of scenario uncertainty in agricultural inputs on life cycle greenhouse gas emissions from agriculture production systems: the case of chemical fertilizers in Japan. Journal of Cleaner Production. 73:109–115.

[56] Li J., Lu T., Yi X., An M., & Hao R. (2024b). Energy systems capacity planning under high renewable penetration considering concentrating solar power. Sustainable Energy Technologies and Assessments, 64, 103671.

[57] Zhang K., Liu Q., Qian H., Xiang B., Cui Q., Zhou J., et al. (2021). EATN: An efficient adaptive transfer network for aspect-level sentiment analysis. IEEE Transactions on Knowledge and Data Engineering, 35(1), 377–389.

[58] Kongshaug G. (1998) Energy consumption and greenhouse gas emissions in fertilizer production. In: IFA Technical Conference, Marrakech, Morocco.

[59] Ekvall T. (2019). Attributional and consequential life cycle assessment. Sustainability assessment at the 21th century. IntechOpen. 10.5772/intechopen.89202.

