# Supplementary material for: Assessing the energy load and environmental footprint of potash fertilizer production in Iran
Source: PLoS One. 2024 Nov 7;19(11):e0313129. doi: 10.1371/journal.pone.0313129 (PMC11542807; doi:10.1371/journal.pone.0313129)
Supplement: S1 Nomenclature — (DOCX) [file pone.0313129.s003.docx]

| **Nomenclature** | | | |
| --- | --- | --- | --- |
| KCL | Potassium Chloride | LCI | Life Cycle Inventory |
| K_2_SO_4_ | Potassium Sulfate | VOC | Volatile Organic Compound |
| GHG | Greenhouse Gas | CLCA | Consequential Life Cycle Assessment |
| LCA | Life Cycle Assessment | LCIA | Life Cycle Impact Assessment |
| AD | Abiotic Depletion | EP | Eutrophication |
| AC | Acidification | GWP | Global Warming Potential |
| OLD | Ozone Layer Depletion | HT | Human Toxicity |
| FE | Fresh water aquatic Ecotoxicity | ME | Marine Aquatic Ecotoxicity |
| TE | Terrestrial Ecotoxicity | PO | Photochemical Oxidation |
